# Medical electives: exploring the determinants of placement and access variables between 2010 and 2016 at the University of Auckland

**DOI:** 10.1186/s12909-019-1784-7

**Published:** 2019-10-29

**Authors:** Judith McCool, Elana Curtis, Andrew D. MacCormick, Alana Cavadino, Michelle Smith, Warwick Bagg

**Affiliations:** 1 Epidemiology and Biostatistics, School of Population Health, Faculty of Medical and Health Sciences, University of Auckland, Auckland, New Zealand; 2 Te Kupenga Hauora Māori, Faculty of Medical and Health Sciences, University of Auckland, Auckland, New Zealand; 3 Medical Programme Directorate, Faculty of Medical and Health Sciences, University of Auckland, Auckland, New Zealand

**Keywords:** Medical education, Elective, Access, Patterns

## Abstract

**Background:**

Medical electives undertaken during sixth year at medical school provide an opportunity for students to work in an overseas or New Zealand health facility to gain exposure to a health system outside their training facility. Previous work suggests that the elective experience can be profound, exposing global health inequities, or influencing future career decisions. This study assessed patterns within elective choice by students’ socio demographic and programme entry characteristics.

**Methods:**

A retrospective analysis of student elective records from 2010 to 2016 was undertaken using a Kaupapa Māori research framework, an approach which prioritises positive benefits for Māori (and Pacific) participants and communities. A descriptive analysis of routinely collected de-identified aggregate secondary data included demographic variables (gender, age group, ethnicity, secondary school decile, year and route of entry), and elective site. Route of entry (into medical school) is via general, MAPAS (Māori and Pacific Admissions Scheme) and RRS (Regional and Rural Scheme). Multivariable logistic regression analysis determined the odd ratios for predictors of going overseas for elective and electives taking place in a “High” (HIC) compared to “Low- and middle-income countries” (LMIC).

**Results:**

Of the 1101 students who undertook an elective (2010–2016) the majority undertook their elective overseas; the majority spent their elective within a high-income country. Age (younger), route of entry (general) and high school decile (high) were associated with going overseas for an elective. Within the MAPAS cohort, Pacific students were more likely (than Māori) were to go overseas for their elective; Māori students were more likely to spend their elective in a HIC.

**Conclusion:**

The medical elective holds an important, pivotal opportunity for medical students to expand their clinical, professional and cultural competency. Our results suggest that targeted support may be necessary to ensure equitable access, particularly for MAPAS students the benefit of an overseas elective.

## Background

Medical electives are an important part of learning within contemporary medical school programmes [1–3]. Variously described, the elective reflects a long held belief in the value of direct exposure to alternative models of health care delivery and with this, the opportunities for personal and professional development. From this perspective, the elective could perform as an agent of change for medical students. Some argue that it may promote a transition to technically sound, socially responsible, culturally competant graduates well positioned to respond to contemporary health care challenges [4].

The University of Auckland (UoA) offers the elective during the final (sixth) year of medical training. Students self-select a country, institution and specialisation of preference for their 8-week elective [1, 2]. Although there is elective planning support provided by a team of elective advisors, the choice of location is entirely student driven. Previous analysis of elective site preferences conducted at the University of Auckland in 2015 found that the majority of students opted to spend their elective in a low or middle income (LMIC) [3]. This work suggested that elective site might reflect personal and professional goals alongside stated elective aims for the training institution. For the UoA, these include experiencing medical practice in other countries, the opportunity to extend and consolidate clinical knowledge, consider future career decisions, to encourage, initiate and build social accountability and responsibility [3]. These reflect the educational aspirations that underpin elective programmes in Aotearoa New Zealand (NZ) as in other countries [4].

Previous work describes the intrinsic benefits for students undertaking an international medical elective [5], including the potential for this experience to shape future career trajectories [4–8], exposure to global health challenges [3, 5, 9], including health care delivery in low resourced settings.

Studies investigating the ethical consideration of medical and global health electives have emerged, with a series of ethical guidelines developed and in some institutions, applied [1, 10]. Among the concerns about the risks associated the overseas elective, including for the student and host community [1, 11] and the varied benefits [5, 12]. The issue of equity of access to participate in the elective experience has been relatively under-researched.

The aim of this study was to examine whether there are significant and meaningful patterns that can characterise elective placements by students’ socio demographic and programme entry characteristics. This is of interest because the elective carries with it a burden of cost to the student and is reliant upon students making their own arrangements, with advisory support, to confirm elective institution site and supervision.

Many universities strive to enrol a student population that matches the population demographic that graduate doctors will serve. At the University of Auckland the medical programme aims to admit 30% Māori and Pacific students via the Māori and Pacific Admission Scheme (MAPAS), and 20% of students from regional or rural backgrounds via Regional Rural Admissions Scheme (RRAS) [13]. Students admitted through MAPAS have verified indigenous Māori and Pacific ancestry and are New Zealand Citizens or permanent residents [14]. The ongoing effects of colonisation and racism contribute to the over representation of Māori and Pacific peoples within the most deprived deciles within New Zealand [15]. This pattern is also seen for students admitted under MAPAS who are more likely to have attended a low-medium school decile (proxy for lower SES) compared to general entry and rural and regional admissions students (RRAS) [16]. Given this context, we hypothesise that MAPAS students may have limited access to financial resources to support an overseas elective or, if they do travel overseas, may be more likely to spend their elective in low or middle-income countries rather than a high-income country. This has implications in terms of access to specialised (including technology driven) elective options. Moreover, students who have dependants may also face greater barriers to accessing an international elective. In addition to SES, it is possible that there are differences for MAPAS and RRAS students in respect to their access to social and professional networks that may influence elective access/type. Māori and Pacific students are more likely to be first in family to undertake tertiary study and therefore may have fewer professional connections [17]. Monitoring access to medical education opportunities is an essential component of building an equity oriented and empowered health workforce.

## Method

### Aim

To describe elective placement patterns according to demographic and route of entry to the medical programme (e.g. general, MAPAS and RRAS) controlling for covariates including age and gender.

To describe the elective placements undertaken by students within the MAPAS cohort, controlling for covariates including age and gender.

### Study design

This study undertook a retrospective analysis of student elective records from 2010 to 2016 within a Kaupapa Māori research framework [18].

Kaupapa Māori Research.

A Kaupapa Māori Research (KMR) framework was used to inform all aspects of the study design including the framing of the research question, the approach to obtaining and using quantitative data and data interpretation [18, 19]. This approach includes: a commitment to ensuring that the research outputs will have positive benefits for Māori (and Pacific) participants and communities; an explicit challenge to reject ‘ victim blame’ and ‘ cultural deficit’ analyses when interpreting data [20]; and ensuring that any recommendations made from the research aim to facilitate academic benefits for participants. A commitment to investigating the patterning of elective usage between MAPAS and other routes of entry as well as within the MAPAS cohort also reflects the KMR positioning of this work. This broad approach is expected to provide benefit for all study participants.

### Sample

In September 2017, we obtained multiple administration data sources from the UoA to investigate patterns in elective placements. Students undertaking a Bachelor of Medicine, Bachelor of Surgery (MBChB) are required to undertake an 8-week medical elective either overseas or in NZ. On completion of the elective, students are required to provide a comprehensive report on their elective experience, including information on country institution, and sub-specialisation, in addition details on the elective experiences on medical, personal and professional development. All elective reports are accessible via an online secure database for staff and students to access for future reference when planning their elective. All reports are reviewed and approved for appropriateness and confidentiality. These data included records for all UoA medical students who undertook an elective during the period from 1 January 2010 to 31 December 2016. Enrolment data via the centralised Student Services Online (SSO) dataset were assessed to gain demographic data (gender, date of birth, ethnicity group, route of entry into the medical programme and secondary school economic status). Elective placement information (country and institution visited, and speciality) was obtained through a pre-departure elective database. The two databases were matched using a unique student ID number. Where possible, missing information was imputed using the individual student’s medical elective report, which are freely available online. Age was included based on the assumption that older aged students (25 and older) may have additional financial pressures and family commitment, which may influence elective destination.

### Data preparation

Duplicates were combined into one entry, and cases where all key demographic information was missing (8 cases) were removed from further analysis.

#### Demographic variables

Previously attended secondary school decile was used as an indicator of socioeconomic status. Secondary schools were classified as either high (decile 9–10), medium (deciles 4–8) or low decile (deciles 1–3), or as an overseas secondary school. Ethnicity from SSO was pre-categorised into Level 1 aggregate ethnic groupings prioritised as either Māori, Pacific, Asian, Other minority or NZ European [21]. Route of entry into the medical programme was defined according to three categories; General (the bulk of medical entrants), MAPAS (Māori and Pacific Admission Scheme), and RRAS (Regional Rural Admission Scheme) [13]. All three routes of entry allow for undergraduate applicants (who have completed the first year of the BHSc or BSc (Biomedical Sciences) degree at the UoA) and graduate applicants (who have completed a degree at a NZ university within 5 years of application). International students were not included in the analysis due to small sample size and potential for different elective usage given their international status (with hypothesis they may be more likely to return to their home country). Age at the beginning of the elective year was calculated, using birth date information, creating two categories (20–24 years old, and over 25 years). Age was included based on the assumption that older aged (25 and older) students may have additional financial pressures and family commitments, which may influence elective destination.

#### Country variables

We categorised elective destinations into two categories; remaining in NZ, going overseas. Students who split their time between overseas and NZ (*n* = 28) were excluded as the outcome variable was going overseas for the entire elective. In the case of those students who went overseas for their entire elective, we further grouped the country destinations by country income into two country economic categories; high-income countries (HIC), upper and lower middle and low-income countries (LMICs). Country income level was created using data from the latest data available (2016) from the World Bank World Development Indicators using the Gross National Income (GNI) per capita [22].

### Statistical analysis

A descriptive analysis conducted with de-identified aggregate secondary data using Stata v15. Frequencies and percentages compared demographic variables (gender, age group, ethnicity, secondary school decile, year and route of entry), and country variables (country income). Associations between variables were assessed using the Pearson chi-square tests for categorical outcomes and the Kruskall-Wallis test for the ordinal outcome of country income level (low, lower-middle, upper-middle or high). Multivariable logistic regression analysis determined the odd ratios for predictors of going overseas for elective. Ordinal regression for country income level failed to meet the proportional odds assumption; we therefore used logistic regression to determine the odds of overseas electives taking place in a “High-income country (HIC)”, compared to the combined category of “Low, Lower-Middle, and Upper-Middle income countries” (LMIC).

Prior to performing logistic regressions, we assessed the association between the variables. Categories within route of entry and ethnicity variables were often highly correlated, indicating a high level of multi-collinearity between the two variables. Ethnicity was therefore not included in the logistic regression models. Route of entry was included as a key independent variable in the analysis as it is hypothesised to be associated with elective placements. We also assessed potential effect modification of all variables in the model by route of entry, by including each interaction term in the model one at a time. This was done to test the hypothesis that the predictors of going overseas for elective and of country income level of overseas elective might vary according to the route of entry. Where significant interactions were present, results were presented separately for each route of entry. If no significant interaction were present, we assessed the MAPAS sample independently to assess any significant differences across the independent variables. This approach is consistent with a Kaupapa Māori methodology whereby independent analyses for Maori and Pacific participants are conducted for explicit representation in the analyses.

This study was approved by the UoA Human Ethics Committee on 30th August 2017 for 3 years. Reference number 019708.

## Results

### Sample characteristics

In total, 1101 student records were used in this analysis. Table 1 presents the demographic characteristics of the sample, according to both route of entry and total sample. Overall students who undertook an elective were predominately aged between 20 and 24 years (*n* = 692, 62.9%), with a mean age of 25 years. Students had mostly attended a medium (*n* = 377, 34.2%) or high decile (*n* = 566, 51.4%) secondary school in New Zealand (a proxy for higher SES) and were predominantly of either Asian (*n* = 365, 33.1%) or NZ European ethnicity (*n* = 466, 42.3%). School decile is used as an indicator of deprivation within the immediate surrounding neighbourhood of the school [23] . Students primarily entered the UoA medical programme through the general entry route (*n* = 748, 67.9%). In unadjusted/univariate analysis, sex, age, secondary school decile, ethnicity were all correlated with route of entry (*p* < 0.01 for all comparisons. There was no association (*p* > 0.05) between year of elective and route of entry.

**Table 1:** Demographics of 1101 University of Auckland medical programme elective students according to route of entry, 2010–2016

Variable	Category	General *N* = 748 (67%)	MAPAS *N* = 212 (19.3%)	RRAS *N* = 141 (3.8%)	Total *N* (%)	*P*-value^a^
Sex	Male	377 (50.4)	84 (39.6)	56 (39.7)	517 (46.9)	
	Female	371 (49.6)	128 (60.4)	85 (60.3)	584 (53.0)	0.004
Age	20–24	507 (67.8)	106 (50.0)	79 (56.0)	692 (62.9)	
	25+	241 (32.2)	106 (50.0)	62 (44.0)	409 (37.1)	< 0.001
Secondary school attended decile^b^	Low (1–3)	30 (4.0)	61 (29.1)	12 (9.0)	103 (9.4)	
	Medium (4–8)	219 (29.4)	75 (35.7)	83 (61.9)	377 (34.2)	
	High (9–10)	464 (62.4)	67 (31.9)	35 (26.1)	566 (51.4)	
	Overseas schooling	31 (4.2)	7 (3.3)	4 (3.0)	42 (3.8)	< 0.001
	Missing				*13 (1.2)*	
Ethnicity	NZ European	347 (47.0)	–	119 (85.6)	466 (42.3)	
	Māori	9 (1.2)	128 (60.7)	1 (0.7)	138 (12.5)	
	Pacific	3 (0.8)	83 (39.3)	1 (0.7)	90 (8.2)	
	Asian	348 (47.1)	–	17 (12.2)	365 (33.1)	
	Other	29 (3.9)	–	1 (0.7)	30 (2.7)	< 0.001
	Missing				*12 (1.1)*	
Year of elective	2010	80 (10.7)	20 (9.4)	24 (17.0)	124 (11.2)	
	2011	98 (13.1)	21 (9.9)	19 (13.5)	138 (12.5)	
	2012	100 (13.4)	24 (11.3)	11 (7.8)	135 (12.3)	
	2013	108 (14.4)	32 (15.1)	23 (16.3)	163 (14.8)	
	2014	114 (15.2)	33 (15.6)	19 (13.5)	166 (15.1)	
	2015	123 (16.4)	36 (17.0)	18 (12.8)	177 (16.1)	
	2016	125 (16.7)	46 (21.7)	27 (19.1)	189 (17.9)	0.32

^a^*P*-value from chi-square test for differences between each variable and route of entry (excluding missing values)

^b^Only applies to those who went to secondary school in NZ

The majority of students spent their elective at an overseas institution (*n* = 861, 78.2%) (Table 2). Of those students who went on an overseas elective, the majority spent their elective within a high-income country (*n* = 422, 48.6%), with the United Kingdom being the most frequented country (*n* = 149, 17.3%). In the unadjusted/univariates analysis, going overseas for elective was correlated with route of entry (*p* = 0.001). There was no association (*p* > 0.05) between income level of overseas placement country with route of entry.

**Table 2:** Summary of University of Auckland medical programme elective placements for 1101 students according to route of entry, 2010–2016

Variable	Category	General *N* = 748 (67%)	MAPAS *N* = 212 (19.3%)	RRAS *N* = 141 (3.8%)	Total N (%)	*P*-value^a^
Placement location	New Zealand	124 (17.0)	59 (28.1)	34 (24.6)	217 (19.7)	
	Overseas	606 (83.0)	151 (71.9)	104 (75.4)	861 (78.2)	0.001
Income level of placement country^b^	Low, Lower-Middle or upper-middle income country (LMIC)	276 (46.7)	76 (55.5)	53 (53.5)	405 (47.0)	
	High Income Country (HIC)	315 (53.3)	61 (44.5)	46 (46.5)	422 (49.0)	0.11
	*Missing*				*34 (4.0)*	

^a^*P*-value from chi-square test for differences between each variable and route of entry (excluding missing values)

^b^Only applies to those taking an overseas placement

### Predictors of going overseas for medical elective

In univariate analyses, age (χ^2^(1) =54.1, *P* < 0.001), ethnicity (χ^2^(4) = 17.8, *P* = 0.001), route of entry (χ^2^(2) = 14.5, *P* = 0.001) and secondary school decile (χ^2^(3) = 11.2, *P* = 0.01) were all associated with going overseas for elective. Sex (*P* = 0.17) and year of elective (*P* = 0.59) were not significantly associated with the students choice to go overseas for their elective.

Table 3 displays estimates from a multivariable logistic regression analysis assessing predictors of students going overseas for their entire medical elective. Age was a significant predictor of going overseas, with students aged 25 years and older being 64% less likely (OR = 0.36, 95%CI 0.26–0.49) to go overseas than younger students (20–24-year olds). Students in the MAPAS and RRAS routes were less likely then general entry students to go overseas for their elective; however, these differences were attenuated (*P* = 0.05 and *P* = 0.07, respectively) after adjusting for age, gender, secondary school decile and year of elective. There were no significant interactions between route of entry and other variables in the model.

**Table 3:** Predictors of going overseas for elective from multivariable logistic regression model

Predictors	Category	OR	CI (95%)	*P*-value
Age	< 25 years old	Reference		
	25+ years old	0.36	0.26–0.49	< 0.001^*^
Route	General	Reference		
	MAPAS	0.67	0.45–1.00	0.05
	RRAS	0.65	0.41–1.03	0.07
Sex	Male	Reference		
	Female	0.94	0.70–1.27	0.45
Year of elective	Per year	0.99	0.92–1.08	0.94
Secondary school decile	High (9–10)	Reference		
	Medium (4–8)	1.07	0.75–1.54	0.37
	Low (1–3)	0.78	0.46–1.33	0.70
	Overseas schooling	0.83	0.39–1.73	0.89
χ^2^(10)	61.24	< 0.001		
Pseudo *R*^2a^	0.06			
N	1065			

The dependent variable in this analysis is country location, with 0 = Remaining in New Zealand and 1 = Going overseas for entire elective

*CI* confidence interval, *OR* odds ratio

* *P* <  0.001

^a^McFadden’s *R*^2^

Although there were no significant interactions of route of entry with other variables, a main hypothesis of this study was to assess predictors of going overseas for elective. We assessed for MAPAS students separately (as well as within the main model) to determine if there are any significant within group differences in uptake of an overseas versus domestic elective and country placement (Table 4). Age remained a strong predictor of going overseas in MAPAS students (*P* = 0.003) after adjusting for sex, year of elective, secondary school decile and ethnicity. Within MAPAS, Pacific students were over 2.5 times more likely to go overseas for their elective than Māori students (OR = 2.63, 95%CI 1.27–5.44).

**Table 4:** Predictors of going overseas for elective in MAPAS students only

Predictors	Category	OR	CI (95%)	*P*-value
Age	< 25 years old	Reference		
	25+ years old	0.36	0.18–0.70	0.003^*^
Ethnicity	Māori	Reference		
	Pacific	2.63	1.27–5.44	0.009^*^
Sex	Male	Reference		
	Female	1.91	0.98–3.71	0.06
Year of elective	Per year	1.02	0.86–1.20	0.83
Secondary school decile	High (9–10)	Reference		
	Medium (4–8)	1.24	0.55–2.80	0.61
	Low (1–3)	0.72	0.32–1.63	0.43
	Overseas schooling^b^	–	–	–
χ^2^(10)	21.72	*P* < 0.001		
Pseudo *R*^2a^	0.09			
N	200			

The dependent variable in this analysis is country location, with 0 = Remaining in New Zealand and 1 = Going overseas for entire elective

*CI* confidence interval, *OR* odds ratio

^*^P <  0.01

^a^McFadden’s *R*^2^

^b^*n* = 7 MAPAS students with overseas schooling were dropped from the model as they all went overseas for their elective (i.e. predict the outcome perfectly)

### Predictors of country income level of overseas elective

The Kruskall-Wallis test indicated significant associations between income level of the country visited for overseas elective (low, lower-middle, upper-middle or high) and the students’ ethnicity (χ^2^(4) = 12.1, *P* = 0.02) and year of elective (χ^2^(6) = 23.0, *P* = 0.0008).

For a multivariable logistic regression analysis, we considered predictors of overseas elective taking place in a HIC versus LMIC. There was a significant interaction between age and route of entry (*p* < 0.005); we therefore present the results from a logistic regression in each route of entry separately in Table 5. There were no significant interactions between route of entry and other variables in the model. After adjusting for ethnicity, sex, year of entry elective and secondary school decile, age did not have a significant influence on whether the general route students opted for a HIC or LMIC. For MAPAS and RRAS students, however, there was an opposing effect of age on the overseas elective country income level; when compared to younger students, those aged 25 and older in RRAS were more likely to go to a HIC whereas the older MAPAS students were less likely to go to a HIC. Within MAPAS, Pacific students were less likely than Māori students, to go to a HIC for their overseas elective. The odds of students choosing a HIC increased over the study period (2010 to 2016); for general route of entry students there was an average annual increase of 12% per year, but this effect was not statistically significant amongst MAPAS or RRAS students. Secondary school decile and sex were not significant predictors of going to a HIC after adjusting for age, ethnicity and year of elective (Table 5).

**Table 5:** Predictors of country income level of overseas elective, stratified by route of entry

		General Route			MAPAS Route			RRAS Route		
Predictors	Category	OR	CI (95%)	*P*-value	OR	CI (95%)	*P*-value	OR	CI (95%)	*P*-value
Age	< 25 years	Reference			Reference			Reference		
	≥25 years	1.25	0.75–1.85	0.26	0.37	0.17–0.80	0.01^*^	4.36	1.62–11.72	0.004^**^
Ethnicity	NZ European	Reference			–	–	–	Reference		
	Māori	0.53	0.09–3.06	0.48	Reference			–	*–*	*–*
	Pacific	0.31	0.03–2.90	0.31	0.29	0.13–0.63	0.002^**^	–	*–*	*–*
	Asian	1.43	1.01–2.04	0.05	–	–	–	–	*–*	*–*
	Other minority	1.32	0.55–3.17	0.53	–	–	–	0.51^b^	0.12–2.10	0.35
Sex	Male	Reference			Reference			Reference		
	Female	0.81	0.58–1.14	0.23	0.93	0.42–2.05	0.86	1.16	0.43–3.06	0.76
Year of elective	Per year	1.12	1.03–1.23	0.008^**^	1.06	0.87–1.28	0.58	1.05	0.85–1.30	0.66
Secondary School decile	High (9–10)	Reference			Reference			Reference		
	Medium (4–8)	0.66	0.45–0.95	0.03^*^	1.26	0.51–3.09	0.61	1.31	0.39–3.26	0.82
	Low (1–3)	0.72	0.30–1.70	0.45	1.35	0.50–3.65	0.55	0.40	0.07–2.30	0.31
	Overseas schooling	0.82	0.32–2.68	0.88	- ^c^	–	–	- ^c^	–	–
χ^2^(10)			22.84	0.01		17.54	0.008		12.39	0.05
Pseudo *R*^2a^			0.03			0.10			0.10	
N			581			131			88	

*CI* confidence interval, *OR* odds ratio

The dependent variable in this analysis is overseas electives county income levels, with 0 = Low, Lower-Middle, and Upper-Middle Income Countries and 1 = High Income Countries

^*^P < 0.05; ^**^P < 0.01

^a^McFadden’s *R*^2^

^b^For analysis in RRAS route only, the Māori (n = 1), Pacific (*n* = 1), Asian (*n* = 17) and other minorities (n = 1) were grouped together due to small numbers

^c^*n* = 5 MAPAS and *n* = 3 RRAS students with overseas schooling were dropped from these models as they all went to a Low, Lower-Middle or Upper-Middle Income Country for their elective (i.e. predict the outcome perfectly)

## Discussion

The medical elective programme is an established, important component of contemporary medical training. Systemic differences in where students undertake their elective could lead to socio-demographic groups accessing different experiences during the elective programme. These differences may contribute to training outcomes, including choice of specialisation and equity within the health workforce. Assessment of the benefits of the elective programme on student training is via a written report (pass / fail or distinction). Consistent with other programmes, the longer- term outcomes of the elective are unclear.

In our analysis, we identified several significant differences in medical elective placement patterns in terms of route of entry, age, gender, and ethnicity. Specifically, our analysis indicates that the majority of students chose to go overseas for their elective (despite the option of staying in NZ). Among those students who went overseas, almost half (49%) undertook their elective in a medical or health care facility in a high-income country (HIC). When stratified by route of entry, the proportion of general entry students who undertook their elective in a HIC increased between 2010 and 2016. This effect was statistically significant for general entry, but not for MAPAS/RRAS (see Table 5).

As expected, age plays a significant role in whether a student will go overseas for their elective, with older students more likely to remain in New Zealand for their entire elective. One explanation for this effect may be due to students who are 25 years and older having increased responsibilities outside of their course work, such as family commitments. Another explanation may be that older students may have already experienced overseas travel and not be seeking these opportunities during their medical training to the same extent as general entry students. However, without further evidence, these reasons remain speculative.

Within the MAPAS cohort, Pacific students were more likely than Māori students to go overseas, albeit to a LMIC. A possible explanation may be that the elective provides an opportunity for predominantly NZ-born Pacific students to return to or visit their Pacific island country of ancestry, although we do not have data to confirm this. In either case, we are cautious about applying assumptions to the relative benefits from a local or LMIC based elective on value added to student training. Similarly, the value of an elective based in a HIC may offer students in terms of training opportunities. What are observing, anecdotally, is a strategic approach to electives driven by factors including cost, perceived value in terms of practical clinical skill building, and testing future career options. We have not assessed implicit drivers to choice of elective placement; this data are captured in terms of elective objectives stated in the application for an elective [24].

This analysis has several limitations. Due to the use of secondary data, we may not have captured a key predictor of elective selection, as indicated by the small pseudo *R*^2^ values in both logistic regression models. This indicates a need for a further exploratory research (using qualitative methods) which could identify other key influencers over student’s elective decisions. The use of secondary data meant we were unable to gather detail on whether the students worked in a public or private facility. This would provide more meaningful information in addition to HIC or LMIC country based elective (for example, some may work in a private hospital within a LMIC or a public funded hospital in a HIC). We also were unable to account for student’s access to financial support, a likely factor in the decision to undertake an overseas or domestic elective.

The medical elective continues to hold an important place in the training of medical students within the UoA because of the opportunity it provides for consolidating and extending clinical, professional, and personal development. This may include the development of an awareness of the drivers to the determinants of health and diversity in health care delivery. For many students, it is their first independent overseas travel, for others, a chance to observe or work alongside internationally respected clinicians or appreciate health care delivery in low resourced settings. Elective destinations and speciality focus while on elective were patterned by factors such as age, decile ranking of school and route of entry in to the medical programme. It was important to identify whether there are significant differences between the General, RRAS and the MAPAS cohorts, with the view to ensuring equitable access to the ‘hidden’ benefits that the elective offers to the medical training programme [12]. These may include a desire to engage with a new culture and language or experience living independently or other aspects, such as a self-awareness and confidence building. This study cannot conclude on the individual drivers behind elective choice as this was not formally examined. Therefore, it remains important to examine the rationale for student elective choice directly and whether there are any differences for Māori and Pacific students compared to non-Māori non-Pacific students.

It is important to recognise that there may be unintended risks associated with the medical elective such as ‘cultural voyeurism’ or ‘clinical tourism’ that potentially reinforces negative stereotypes or essentialises ‘other’ cultures [25, 26]. Levi (2008) [27] stresses that preparation for clinical global experiences must be thoughtful, directed, and include opportunities for students to reflect on their own values, biases and beliefs about cultural differences. This study has not been designed to specifically investigate the issue of widen participation within the medical programme, rather the issue of equitable access to additional opportunities embedded with the medical programme, such as an international elective. Globally, the issue of equity and uncompromised access to medical training opportunities remains a challenge [28]. These issues and further research is recommended to ensure that the medical elective supports the development of culturally-safe medical practitioners [27, 29].

## Conclusion

The elective programme endeavours to provide equitable opportunities for all medical students to participate in the globalised world of medicine. Ensuring equitable access for MAPAS students to engage with the intended outcomes of the elective programme remains a priority for ensuring a diverse, globally connected, culturally-safe and professionally competent health workforce.

## Data Availability

The data that support the findings of this study are available from The University of Auckland Medical Programme Directorate but restrictions apply to the availability of these data, which were used under license for the current study, and so are not publicly available. Data are however available from the authors upon reasonable request and with permission of the University of Auckland Medical Programme Directorate.

## Abbreviations

BHSc: Bachelor of Health Science
HIC: High-income countries
KMR: Kaupapa Māori Research
LMIC: Low and middle-income countries (LMIC)
MAPAS: Maori and Pasifika Admission Scheme
RRAS: Regional and Rural admissions scheme
UOA: University of Auckland (UoA)
